# 2-Amino-4-methyl­pyridinium 6-carb­oxy­pyridine-2-carboxyl­ate methanol monosolvate

**DOI:** 10.1107/S1600536810050245

**Published:** 2010-12-08

**Authors:** Hossein Aghabozorg, Azadeh Mofidi Rouchi, Masoud Mirzaei, Behrouz Notash

**Affiliations:** a Faculty of Chemistry, Islamic Azad University, North Tehran Branch, Tehran, Iran; b Department of Chemistry, School of Sciences, Ferdowsi University of Mashhad, Mashhad, Iran; c Department of Chemistry, Shahid Beheshti University, G.C., Evin, Tehran 1983963113, Iran

## Abstract

In the title solvated molecular salt, C_6_H_9_N_2_^+^·C_7_H_4_NO_4_^−^·CH_4_O, the pyridine N atom of 2-amino-4-methyl­pyridine is protonated and one carboxyl group of pyridine-2,6-dicarb­oxy­lic acid is deprotonated. The dihedral angles between the –CO_2_ and –COH groups and the pyridine ring are 0.65 (13) and 7.4°. The crystal packing is stabilized by inter­molecular N—H⋯O, O—H⋯O and weak C—H⋯O hydrogen bonds.

## Related literature

For background to proton-transfer compounds, see: Aghabozorg *et al.* (2008 ▶). For related structures, see: Aakeröy *et al.* (1998 ▶); Aghabozorg *et al.* (2006 ▶); Al-Allaf *et al.* (2003 ▶); Fu *et al.* (2005 ▶); Linden *et al.* (2003 ▶); Moghimi *et al.* (2004 ▶); Sheshmani *et al.* (2006 ▶); Thanigaimani *et al.* (2007 ▶).
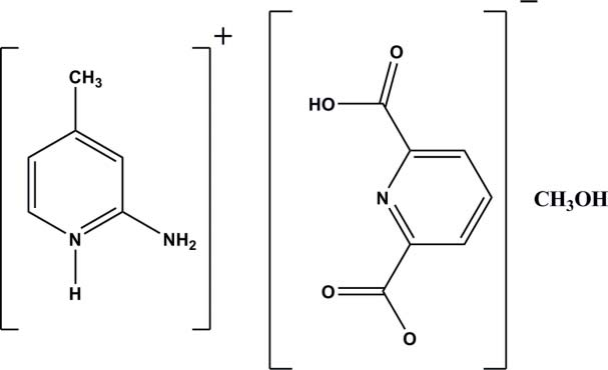


## Experimental

### 

#### Crystal data

C_6_H_9_N_2_^+^·C_7_H_4_NO_4_^−^·CH_4_O
*M* *_r_* = 307.31
Triclinic, 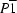

*a* = 7.2191 (14) Å
*b* = 9.5095 (19) Å
*c* = 11.139 (2) Å
α = 94.44 (3)°
β = 99.76 (3)°
γ = 92.50 (3)°
*V* = 750.1 (3) Å^3^
*Z* = 2
Mo *K*α radiation
μ = 0.11 mm^−1^
*T* = 298 K
0.4 × 0.25 × 0.2 mm

#### Data collection

Stoe IPDS II diffractometer
8658 measured reflections
4005 independent reflections
2697 reflections with *I* > 2σ(*I*)
*R*_int_ = 0.058

#### Refinement

*R*[*F* ^2^ > 2σ(*F* ^2^)] = 0.079
*wR*(*F* ^2^) = 0.216
*S* = 1.17
4005 reflections
222 parameters
H atoms treated by a mixture of independent and constrained refinement
Δρ_max_ = 0.44 e Å^−3^
Δρ_min_ = −0.26 e Å^−3^

### 

Data collection: *X-AREA* (Stoe & Cie, 2005 ▶); cell refinement: *X-AREA*; data reduction: *X-AREA*; program(s) used to solve structure: *SHELXS97* (Sheldrick, 2008 ▶); program(s) used to refine structure: *SHELXL97* (Sheldrick, 2008 ▶); molecular graphics: *ORTEP-3 for Windows* (Farrugia, 1997 ▶); software used to prepare material for publication: *WinGX* (Farrugia, 1999 ▶).

## Supplementary Material

Crystal structure: contains datablocks I, global. DOI: 10.1107/S1600536810050245/bt5416sup1.cif

Structure factors: contains datablocks I. DOI: 10.1107/S1600536810050245/bt5416Isup2.hkl

Additional supplementary materials:  crystallographic information; 3D view; checkCIF report

## Figures and Tables

**Table 1:** Hydrogen-bond geometry (Å, °)

*D*—H⋯*A*	*D*—H	H⋯*A*	*D*⋯*A*	*D*—H⋯*A*
C14—H14*C*⋯O2^i^	0.96	2.59	3.488 (5)	157
O5—H5*A*⋯O3^ii^	0.78 (5)	2.02 (5)	2.796 (3)	170 (4)
N3—H3*B*⋯O4^iii^	0.83 (4)	1.95 (4)	2.764 (3)	166 (3)
N3—H3*A*⋯O2^i^	0.87 (4)	2.30 (4)	3.122 (3)	158 (3)
N2—H2⋯O3^iii^	0.85 (3)	1.87 (3)	2.723 (3)	173 (3)
O1—H1⋯O5^iv^	0.87 (4)	1.87 (4)	2.689 (3)	156 (4)

Symmetry codes: (i) 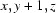; (ii) 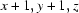; (iii) 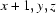; (iv) 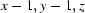.

**Fig. 1.**
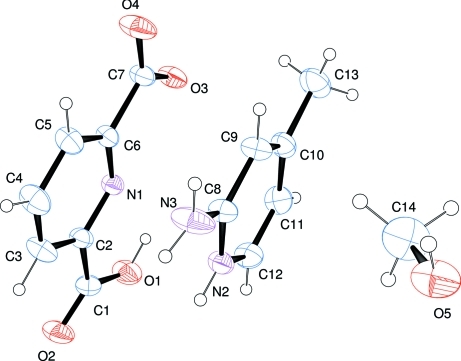
The molecular structure of the title compound with displacement ellipsoids drawn at 30% probability level.

**Fig. 2.**
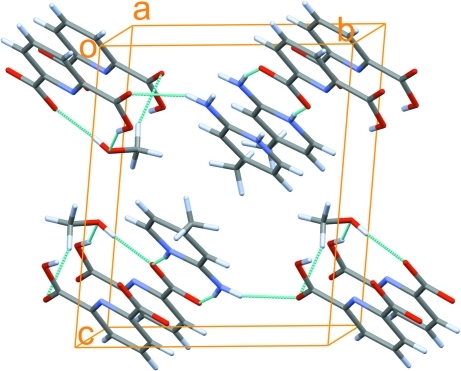
The packing diagram of the title compound. The intermolecular N—H···O, O—H···O and C—H···O hydrogen bonds are shown as blue dashed lines.

## supplementary crystallographic information

### Comment

Continuing the path to synthesize proton transfer compounds, our group have been focused on forming ion pairs between 2,6-pydcH_2_ and various organic bases (Aghabozorg *et al.*, 2008). Due to its flat and symmetric structure and two proton donor sites, 2,6-pydcH_2_ has a potential of constructing supramolecular networks. Proton transfer compounds of 2,6-pydcH_2_ with nitrogen donor molecules such as 2-chloro-benzylamine (Aakeröy *et al.*,1998), piperazine (Aghabozorg *et al.*, 2006 & Sheshmani *et al.*, 2006), phenanthroline (Fu *et al.*, 2005), creatinine (Moghimi *et al.*, 2004) and 2-amino-4,6-dimethoxypyrimidine (Thanigaimani *et al.*, 2007) have been synthesized and characterized by single-crystal X-ray diffraction method. In addition, the formation of monoprotonated 2-amino-4-methylpyridine (2a4mpH) has been reported in several proton transfer systems (Al-Allaf *et al.*, 2003; Linden *et al.* 2003).

The title compound, (2a4mpH)(2,6-pydcH).CH_3_OH, consist of one mono deprotonated 2,6-pydcH_2_ unit, one mono protonated 2a4mp, and one methanol molecule. The asymmetric unit of the title compound is shown in Fig. 1. The title compound, was formed from the reaction between 2,6-pydcH_2_ as a proton donor and 2a4mp as a proton acceptor. There are several N—H···O, O—H···O and weak C—H···O hydrogen bonds, in crystal structure of the title compound (Table 1 & Fig. 2). The crystal structure shows that one of the protons of carboxylic groups has been transferred to N_pyridine_ of 2a4mp. Indeed, the structure formed self-assembled supramolecular network through noncovalent interactions.

### Experimental

The reaction between a solution of 2,6-pydcH_2_ (0.1671 mg, 1 mmol) in 10 ml water and 2a4mp (0.2163 mg, 2 mmol) in 10 ml methanol in 1:2 molar ratios gave block colorless crystals of the title compound after slow evaporation of the solvent at room temperature (m.p: 267).

### Refinement

The hydrogen atoms bonded to N and O were found in a difference Fourier map and refined isotropically. The C—H protons were positioned geometrically and refined as riding atoms with C—H = 0.93 Å and *U*iso(H) = 1.2 *U*eq(C) for aromatic C—H groups and C—H = 0.98 Å and *U*iso(H) = 1.5 *Ueq*(C) for methyl group.

### Crystal data

**Table:**

C_6_H_9_N_2_^+^·C_7_H_4_NO_4_^−^·CH_4_O	*Z* = 2
*M**_r_* = 307.31	*F*(000) = 324.0
Triclinic, *P*1	*D*_x_ = 1.361 Mg m^−^^3^
Hall symbol: -P 1	Mo *K*α radiation, λ = 0.71073 Å
*a* = 7.2191 (14) Å	Cell parameters from 4005 reflections
*b* = 9.5095 (19) Å	θ = 2.2–29.2°
*c* = 11.139 (2) Å	µ = 0.11 mm^−^^1^
α = 94.44 (3)°	*T* = 298 K
β = 99.76 (3)°	Block, colorless
γ = 92.50 (3)°	0.4 × 0.25 × 0.2 mm
*V* = 750.1 (3) Å^3^	

### Data collection

**Table:**

Stoe IPDS II diffractometer	2697 reflections with *I* > 2σ(*I*)
Radiation source: fine-focus sealed tube	*R*_int_ = 0.058
graphite	θ_max_ = 29.2°, θ_min_ = 2.2°
Detector resolution: 0.15 mm pixels mm^-1^	*h* = −9→9
rotation method scans	*k* = −12→13
8658 measured reflections	*l* = −15→15
4005 independent reflections	

### Refinement

**Table:**

Refinement on *F*^2^	Secondary atom site location: difference Fourier map
Least-squares matrix: full	Hydrogen site location: inferred from neighbouring sites
*R*[*F*^2^ > 2σ(*F*^2^)] = 0.079	H atoms treated by a mixture of independent and constrained refinement
*wR*(*F*^2^) = 0.216	*w* = 1/[σ^2^(*F*_o_^2^) + (0.0939*P*)^2^ + 0.144*P*] where *P* = (*F*_o_^2^ + 2*F*_c_^2^)/3
*S* = 1.17	(Δ/σ)_max_ < 0.001
4005 reflections	Δρ_max_ = 0.44 e Å^−^^3^
222 parameters	Δρ_min_ = −0.26 e Å^−^^3^
0 restraints	Extinction correction: *SHELXL97* (Sheldrick, 2008), Fc^*^=kFc[1+0.001xFc^2^λ^3^/sin(2θ)]^-1/4^
Primary atom site location: structure-invariant direct methods	Extinction coefficient: 0.07 (2)

### Special details

**Table:**

Geometry. All e.s.d.'s (except the e.s.d. in the dihedral angle between two l.s. planes) are estimated using the full covariance matrix. The cell e.s.d.'s are taken into account individually in the estimation of e.s.d.'s in distances, angles and torsion angles; correlations between e.s.d.'s in cell parameters are only used when they are defined by crystal symmetry. An approximate (isotropic) treatment of cell e.s.d.'s is used for estimating e.s.d.'s involving l.s. planes.
Refinement. Refinement of *F*^2^ against ALL reflections. The weighted *R*-factor *wR* and goodness of fit *S* are based on *F*^2^, conventional *R*-factors *R* are based on *F*, with *F* set to zero for negative *F*^2^. The threshold expression of *F*^2^ > σ(*F*^2^) is used only for calculating *R*-factors(gt) *etc*. and is not relevant to the choice of reflections for refinement. *R*-factors based on *F*^2^ are statistically about twice as large as those based on *F*, and *R*- factors based on ALL data will be even larger.

### Fractional atomic coordinates and isotropic or equivalent isotropic displacement parameters (Å^2^)

**Table:**

	*x*	*y*	*z*	*U*_iso_*/*U*_eq_	
O1	0.0312 (3)	−0.1514 (2)	0.73074 (19)	0.0679 (6)	
O2	0.2937 (3)	−0.1669 (2)	0.8602 (2)	0.0686 (6)	
O3	−0.3748 (2)	0.20309 (19)	0.76854 (17)	0.0544 (5)	
O4	−0.2965 (3)	0.3911 (2)	0.9022 (2)	0.0730 (7)	
O5	0.7131 (3)	0.9637 (2)	0.6320 (2)	0.0685 (6)	
N1	−0.0374 (3)	0.09454 (18)	0.84467 (16)	0.0381 (4)	
N2	0.2829 (3)	0.3098 (2)	0.70000 (18)	0.0424 (5)	
N3	0.3671 (4)	0.5125 (3)	0.8268 (3)	0.0689 (8)	
C2	0.1276 (3)	0.0410 (2)	0.8845 (2)	0.0415 (5)	
C3	0.2618 (4)	0.1087 (3)	0.9759 (3)	0.0567 (7)	
H3	0.3748	0.0678	1.0017	0.068*	
C4	0.2249 (4)	0.2381 (3)	1.0281 (3)	0.0587 (7)	
H4	0.3135	0.2872	1.0890	0.070*	
C5	0.0548 (4)	0.2936 (2)	0.9886 (2)	0.0501 (6)	
H5	0.0264	0.3806	1.0229	0.060*	
C6	−0.0741 (3)	0.2185 (2)	0.8971 (2)	0.0386 (5)	
C1	0.1592 (4)	−0.1007 (3)	0.8243 (2)	0.0495 (6)	
C7	−0.2648 (3)	0.2756 (2)	0.8524 (2)	0.0455 (5)	
C8	0.2394 (3)	0.4369 (2)	0.7460 (2)	0.0447 (5)	
C9	0.0583 (3)	0.4838 (3)	0.7033 (2)	0.0509 (6)	
H9	0.0248	0.5712	0.7337	0.061*	
C10	−0.0679 (3)	0.4020 (3)	0.6179 (2)	0.0478 (6)	
C11	−0.0140 (4)	0.2706 (3)	0.5725 (2)	0.0545 (6)	
H11	−0.0972	0.2136	0.5138	0.065*	
C12	0.1599 (4)	0.2277 (3)	0.6147 (2)	0.0515 (6)	
H12	0.1953	0.1408	0.5848	0.062*	
C13	−0.2608 (4)	0.4518 (4)	0.5746 (3)	0.0691 (8)	
H13A	−0.3498	0.4075	0.6168	0.104*	
H13B	−0.2964	0.4272	0.4882	0.104*	
H13C	−0.2592	0.5525	0.5910	0.104*	
C14	0.5526 (5)	0.8734 (4)	0.6264 (4)	0.0877 (11)	
H14A	0.5833	0.7775	0.6097	0.132*	
H14B	0.4556	0.8979	0.5625	0.132*	
H14C	0.5088	0.8831	0.7032	0.132*	
H5A	0.700 (6)	1.030 (5)	0.676 (4)	0.097 (14)*	
H2	0.392 (5)	0.282 (3)	0.727 (3)	0.058 (8)*	
H1	−0.058 (6)	−0.094 (4)	0.713 (4)	0.095 (12)*	
H3A	0.338 (5)	0.595 (4)	0.855 (3)	0.084 (11)*	
H3B	0.465 (5)	0.479 (4)	0.861 (3)	0.068 (9)*	

### Atomic displacement parameters (Å^2^)

**Table:**

	*U*^11^	*U*^22^	*U*^33^	*U*^12^	*U*^13^	*U*^23^
O1	0.0737 (14)	0.0578 (11)	0.0673 (13)	0.0256 (10)	0.0023 (10)	−0.0158 (9)
O2	0.0602 (12)	0.0590 (11)	0.0874 (14)	0.0301 (9)	0.0100 (10)	0.0034 (10)
O3	0.0426 (9)	0.0509 (9)	0.0627 (11)	0.0152 (7)	−0.0078 (8)	−0.0096 (8)
O4	0.0609 (12)	0.0584 (11)	0.0882 (15)	0.0281 (9)	−0.0126 (10)	−0.0246 (10)
O5	0.0592 (12)	0.0678 (13)	0.0696 (13)	0.0078 (10)	−0.0027 (10)	−0.0227 (10)
N1	0.0377 (9)	0.0360 (9)	0.0402 (9)	0.0065 (7)	0.0046 (7)	0.0020 (7)
N2	0.0400 (10)	0.0427 (10)	0.0432 (10)	0.0103 (8)	0.0031 (8)	0.0000 (8)
N3	0.0466 (13)	0.0550 (13)	0.0925 (19)	0.0163 (11)	−0.0104 (12)	−0.0305 (13)
C2	0.0390 (11)	0.0390 (10)	0.0482 (12)	0.0102 (8)	0.0089 (9)	0.0069 (9)
C3	0.0380 (12)	0.0537 (14)	0.0738 (17)	0.0107 (10)	−0.0055 (11)	0.0060 (12)
C4	0.0488 (14)	0.0515 (14)	0.0654 (16)	−0.0006 (11)	−0.0137 (12)	−0.0053 (12)
C5	0.0527 (14)	0.0368 (11)	0.0546 (14)	0.0052 (10)	−0.0049 (11)	−0.0040 (10)
C6	0.0381 (11)	0.0355 (10)	0.0408 (11)	0.0061 (8)	0.0020 (8)	0.0031 (8)
C1	0.0512 (14)	0.0451 (12)	0.0546 (14)	0.0161 (10)	0.0137 (11)	0.0014 (10)
C7	0.0418 (12)	0.0420 (11)	0.0506 (13)	0.0132 (9)	0.0021 (9)	−0.0014 (9)
C8	0.0387 (11)	0.0413 (11)	0.0526 (13)	0.0072 (9)	0.0058 (9)	−0.0029 (9)
C9	0.0421 (12)	0.0462 (12)	0.0648 (15)	0.0127 (10)	0.0091 (11)	0.0011 (11)
C10	0.0370 (11)	0.0550 (13)	0.0519 (13)	0.0036 (10)	0.0043 (10)	0.0136 (10)
C11	0.0503 (14)	0.0585 (15)	0.0491 (14)	−0.0037 (11)	−0.0016 (11)	−0.0042 (11)
C12	0.0538 (14)	0.0449 (12)	0.0530 (14)	0.0051 (10)	0.0067 (11)	−0.0077 (10)
C13	0.0419 (14)	0.081 (2)	0.083 (2)	0.0088 (13)	−0.0001 (13)	0.0215 (16)
C14	0.078 (2)	0.076 (2)	0.106 (3)	−0.0007 (18)	0.012 (2)	0.003 (2)

### Geometric parameters (Å, °)

**Table:**

O1—C1	1.315 (3)	C4—C5	1.375 (4)
O1—H1	0.87 (4)	C4—H4	0.9300
O2—C1	1.208 (3)	C5—C6	1.386 (3)
O3—C7	1.257 (3)	C5—H5	0.9300
O4—C7	1.240 (3)	C6—C7	1.520 (3)
O5—C14	1.401 (4)	C8—C9	1.417 (3)
O5—H5A	0.78 (5)	C9—C10	1.367 (4)
N1—C6	1.332 (3)	C9—H9	0.9300
N1—C2	1.335 (3)	C10—C11	1.407 (4)
N2—C8	1.347 (3)	C10—C13	1.504 (4)
N2—C12	1.356 (3)	C11—C12	1.356 (4)
N2—H2	0.85 (3)	C11—H11	0.9300
N3—C8	1.318 (3)	C12—H12	0.9300
N3—H3A	0.87 (4)	C13—H13A	0.9600
N3—H3B	0.83 (4)	C13—H13B	0.9600
C2—C3	1.378 (4)	C13—H13C	0.9600
C2—C1	1.503 (3)	C14—H14A	0.9600
C3—C4	1.376 (4)	C14—H14B	0.9600
C3—H3	0.9300	C14—H14C	0.9600
			
C1—O1—H1	112 (3)	O3—C7—C6	117.74 (19)
C14—O5—H5A	106 (3)	N3—C8—N2	118.9 (2)
C6—N1—C2	118.17 (19)	N3—C8—C9	123.1 (2)
C8—N2—C12	122.1 (2)	N2—C8—C9	118.0 (2)
C8—N2—H2	118 (2)	C10—C9—C8	120.7 (2)
C12—N2—H2	120 (2)	C10—C9—H9	119.6
C8—N3—H3A	118 (2)	C8—C9—H9	119.6
C8—N3—H3B	123 (2)	C9—C10—C11	118.7 (2)
H3A—N3—H3B	118 (3)	C9—C10—C13	120.3 (3)
N1—C2—C3	123.2 (2)	C11—C10—C13	121.0 (3)
N1—C2—C1	115.8 (2)	C12—C11—C10	119.6 (2)
C3—C2—C1	121.0 (2)	C12—C11—H11	120.2
C4—C3—C2	118.5 (2)	C10—C11—H11	120.2
C4—C3—H3	120.8	C11—C12—N2	120.9 (2)
C2—C3—H3	120.8	C11—C12—H12	119.5
C5—C4—C3	118.9 (2)	N2—C12—H12	119.5
C5—C4—H4	120.6	C10—C13—H13A	109.5
C3—C4—H4	120.6	C10—C13—H13B	109.5
C4—C5—C6	119.3 (2)	H13A—C13—H13B	109.5
C4—C5—H5	120.3	C10—C13—H13C	109.5
C6—C5—H5	120.3	H13A—C13—H13C	109.5
N1—C6—C5	122.0 (2)	H13B—C13—H13C	109.5
N1—C6—C7	117.22 (19)	O5—C14—H14A	109.5
C5—C6—C7	120.77 (19)	O5—C14—H14B	109.5
O2—C1—O1	121.0 (2)	H14A—C14—H14B	109.5
O2—C1—C2	122.2 (2)	O5—C14—H14C	109.5
O1—C1—C2	116.8 (2)	H14A—C14—H14C	109.5
O4—C7—O3	125.9 (2)	H14B—C14—H14C	109.5
O4—C7—C6	116.3 (2)		
			
C6—N1—C2—C3	−0.8 (3)	N1—C6—C7—O4	−180.0 (2)
C6—N1—C2—C1	178.48 (19)	C5—C6—C7—O4	0.1 (4)
N1—C2—C3—C4	−0.5 (4)	N1—C6—C7—O3	−0.6 (3)
C1—C2—C3—C4	−179.8 (2)	C5—C6—C7—O3	179.4 (2)
C2—C3—C4—C5	1.1 (4)	C12—N2—C8—N3	−178.6 (3)
C3—C4—C5—C6	−0.4 (4)	C12—N2—C8—C9	0.4 (4)
C2—N1—C6—C5	1.5 (3)	N3—C8—C9—C10	178.9 (3)
C2—N1—C6—C7	−178.5 (2)	N2—C8—C9—C10	0.0 (4)
C4—C5—C6—N1	−0.9 (4)	C8—C9—C10—C11	−0.5 (4)
C4—C5—C6—C7	179.1 (2)	C8—C9—C10—C13	179.1 (2)
N1—C2—C1—O2	−172.4 (2)	C9—C10—C11—C12	0.5 (4)
C3—C2—C1—O2	6.9 (4)	C13—C10—C11—C12	−179.0 (3)
N1—C2—C1—O1	6.6 (3)	C10—C11—C12—N2	−0.1 (4)
C3—C2—C1—O1	−174.1 (2)	C8—N2—C12—C11	−0.3 (4)

### Hydrogen-bond geometry (Å, °)

**Table:**

*D*—H···*A*	*D*—H	H···*A*	*D*···*A*	*D*—H···*A*
C14—H14C···O2^i^	0.96	2.59	3.488 (5)	157.
O5—H5A···O3^ii^	0.78 (5)	2.02 (5)	2.796 (3)	170 (4)
N3—H3B···O4^iii^	0.83 (4)	1.95 (4)	2.764 (3)	166 (3)
N3—H3A···O2^i^	0.87 (4)	2.30 (4)	3.122 (3)	158 (3)
N2—H2···O3^iii^	0.85 (3)	1.87 (3)	2.723 (3)	173 (3)
O1—H1···O5^iv^	0.87 (4)	1.87 (4)	2.689 (3)	156 (4)

Symmetry codes: (i) *x*, *y*+1, *z*; (ii) *x*+1, *y*+1, *z*; (iii) *x*+1, *y*, *z*; (iv) *x*−1, *y*−1, *z*.
